# Pancytopenia: A Rare and Unusual Initial Presentation of Breast Cancer

**DOI:** 10.7759/cureus.4235

**Published:** 2019-03-12

**Authors:** Vishal Jindal, Anannya Patwari, Vineel Bhatlapenumarthi, Ahmad D Siddiqui

**Affiliations:** 1 Internal Medicine, St. Vincent Hospital, Worcester, USA; 2 Hematology and Oncology, St. Vincent Hospital, Shrewsbury, USA

**Keywords:** breast cancer, pancytopenia

## Abstract

Bone marrow metastasis with profound pancytopenia is an extremely uncommon presentation of breast cancer. Advanced breast cancer can frequently metastasize to bone marrow, but bone marrow failure is not typically seen. Very limited data exist regarding the appropriate management of patients with metastatic breast cancer with profound pancytopenia. Our patient’s initial presentation of anemia and thrombocytopenia was a diagnostic dilemma, later confirmed as metastatic breast cancer on bone marrow biopsy. After diagnosis, treatment was another challenge as there are no predefined treatment guidelines for these patients. After the initial hormonal therapy failed, our patient showed a good clinical response to chemotherapy and her platelet count improved to baseline. This dramatic response to chemotherapy is rare. Therefore, this case represents a rare instance of a diagnostic and therapeutic dilemma with unusual clinical response to chemotherapy.

## Introduction

Breast cancer is the most common cancer in women, and it is the most common cause of death in females between age 45 and 55 years in the USA [1]. Advanced cases are generally incurable, but most of the patients generally present in the initial stages of cancer. Around 20% of patients with operable breast cancer relapse, and of those, 70% relapse as distant metastasis [2-3]. Common sites of breast cancer metastasis are bone, liver, brain, lungs, and lymph nodes [4]. Skeletal metastasis is common in breast cancer [5] and generally presents as bony pains, pathological fractures, spinal cord compression, and hypercalcemia [6]. Sometimes palliative surgery or radiation therapy is used to treat pain or impending fracture due to metastasis. Skeletal metastasis is often the result of the infiltration of bone marrow with further progression to the skeletal cortex. Among breast cancer patients, bone marrow infiltration is commonly seen, but profound pancytopenia is extremely rare [7].

## Case presentation

**Figure 1:**
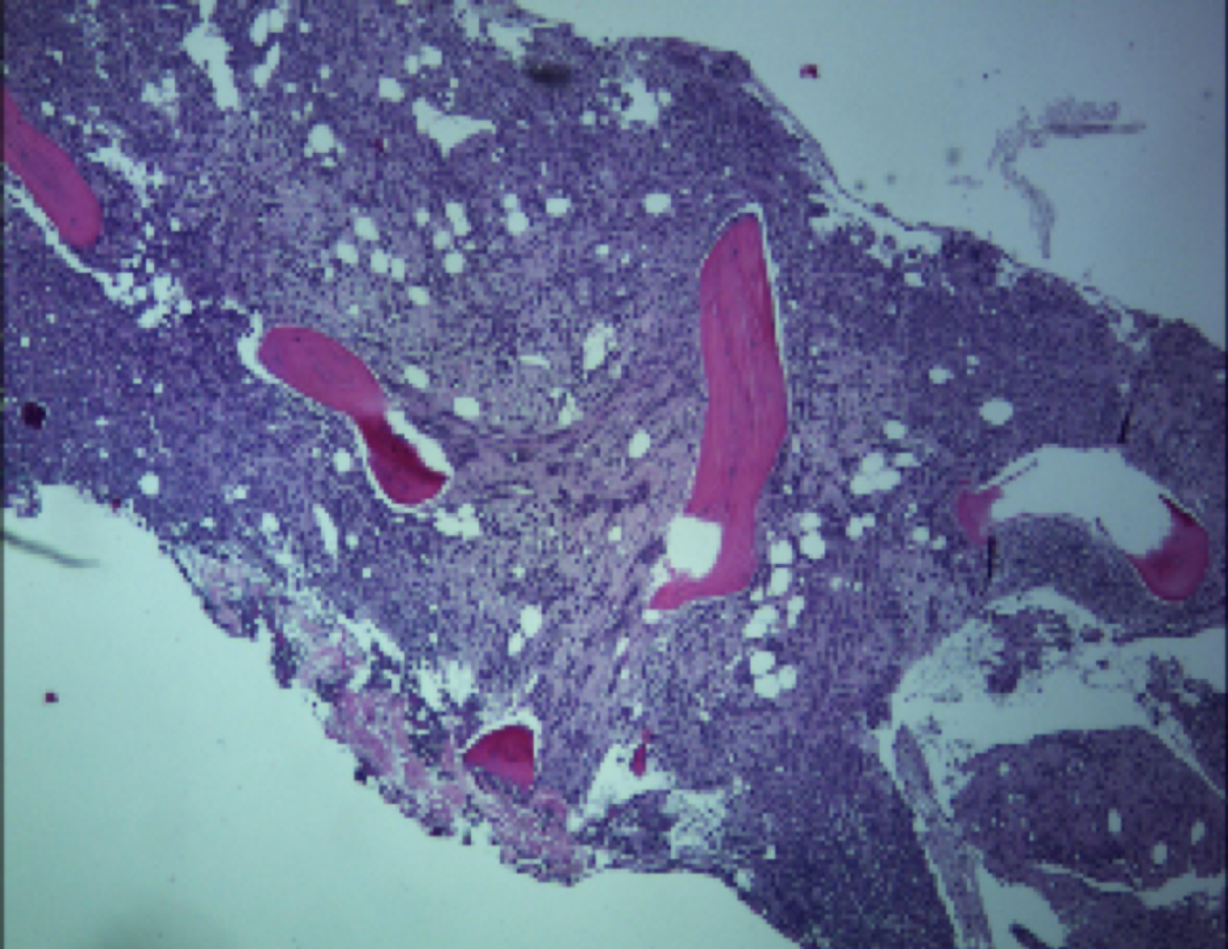
Low-power microscopic image of the bone marrow filled with neoplastic cells.

We present the case of a 56-year-old postmenopausal woman with a past medical history significant for asthma and anxiety disorder who presented with a one-month history of progressive myalgia, dizziness, exertional dyspnea, cough, and fatigue preceded by a prodrome of mild fever and upper respiratory tract infection symptoms. Apart from a recent travel to Peru and a hiking trip in New Hampshire, she denied any other exposures. She does not have any significant family history. Her husband was recently treated for Lyme disease. At the time of admission, her blood work revealed a white blood cell count of 12,000 cells/µL, hemoglobin of 9 g/dL, and platelet count of 22,000 cells/µL. She was started on doxycycline for presumed tick-borne illness, but on treatment, her symptoms continued to worsen. She was evaluated with iron studies, vitamin B12, folate levels, hemolytic panel, liver function test, and serological testing of tick-borne illnesses, all of which yielded unremarkable results. A peripheral smear showed normocytic normochromic red blood cells (RBC). She required packed RBC transfusion for symptomatic anemia. Because of worsening anemia and thrombocytopenia, bone marrow aspiration and biopsy were performed which showed metastatic lobular carcinoma of the breast (Figure 1).

**Figure 2:**
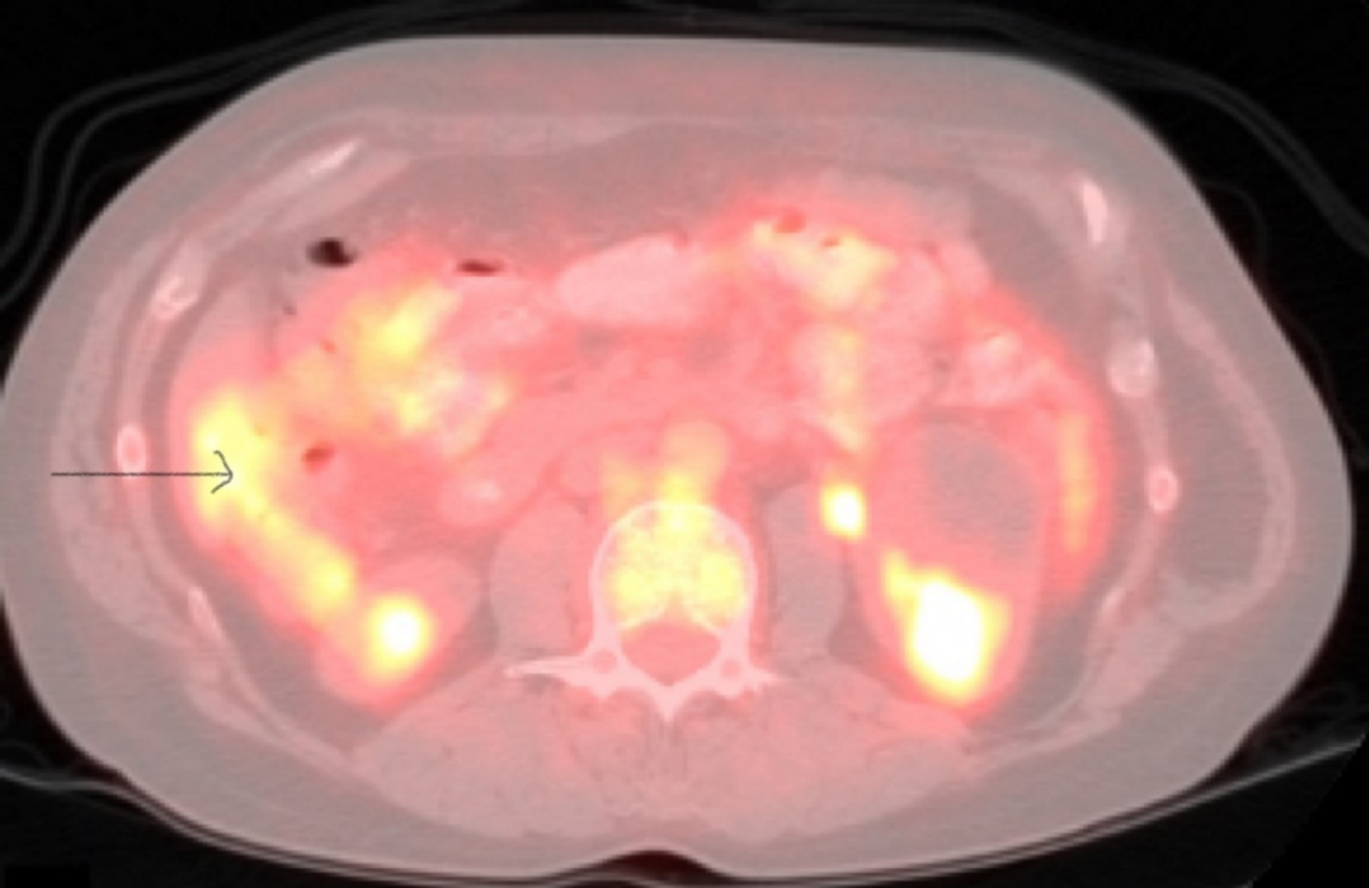
Positron emission tomography/computed tomography (PET/CT) showing the focal medial wall thickening in the cecum.

Immunostains showed that the tumor cells were positive for cytokeratin 7 (CK7), cytokeratin AE1/AE3, estrogen receptor (ER) (about 80%, strong) and negative for cytokeratin 20 (CK20), E-cadherin, thyroid transcription factor 1 (TTF-1), progesterone receptor (PR), and human epidermal growth factor receptor 2 (Her2); this raised suspicion of breast cancer. Breast examination was conducted at that time, and around 2 cm x 2 cm mass was palpated in left breast. A mammogram and breast ultrasound were also obtained, revealing a 1.9 cm x 1.9 cm x 1.3 cm primarily hyperechoic heterogeneous mass with multiple regions of antiparallel hypoechoic spiculated densities with posterior shadowing. To rule out other metastasis, positron emission tomography/computed tomography (PET/CT) scans were obtained (Figure 2) showing focal medial wall thickening in the cecum. We performed a colonoscopy with biopsy which showed metastatic breast cancer. Therefore, our patient was diagnosed with stage IV breast cancer with ER+, PR-, Her2‑.

Our patient was then started on letrozole and palbociclib. Soon after starting the therapy, she developed immune thrombocytopenia and autoimmune hemolytic anemia. The results of her direct antiglobulin test were positive for immunoglobulin G. Therefore, she received steroid therapy which was tapered slowly over two to three months, and her palbociclib and letrozole therapy was discontinued. The patient was started on chemotherapy with capecitabine. She had a good response to this therapy with counts slowly returning to reference range over the next four to six months. Her follow-up scans showed no new metastatic disease, and we noted significant shrinkage of the known breast mass. She tolerated this therapy very well.

## Discussion

In developed countries with established breast cancer screening programs, the most common type of breast cancer presentation is an abnormal screening mammogram. With better diagnostic and early detection modalities, cure rates have improved [8]. Breast cancer commonly metastasizes to bone, liver, and lungs [4]. However, pancytopenia as the herald of breast cancer is very unusual [9]. According to the literature, pancytopenia is caused by alkylating agents and topoisomerase II agents as adjuvant chemotherapy rather than metastatic disease [10]. Some studies also report that the use of growth factors like filgrastim can cause pancytopenia secondary to acute myeloid leukemia or myelodysplastic syndrome [11]. Saito et al. also reported that pancytopenia in a patient with metastatic breast cancer is likely caused due to therapy-induced acute myeloid leukemia [12]. However, in our case, pancytopenia was likely due to bone marrow metastasis-an extremely rare cause according to the literature.

Our patient’s anemia and thrombocytopenia were worrisome, as she already received a blood transfusion. We were also concerned because cytotoxic chemotherapy can cause bone marrow suppression, which can accentuate pancytopenia and can even lead to lethal bleeding complications. Our patient’s dramatic clinical response to chemotherapy with normalization of platelet count is highly unusual. Capecitabine successfully treated bone marrow metastasis without the need for further RBC or platelet transfusion. There are few reports mentioned in the literature regarding the management of bone marrow metastasis with low-dose capecitabine, trastuzumab monotherapy, and endocrine therapy [13-16]. We initially tried hormonal therapy in our patient, but she did not show good response. Therefore, we shifted to capecitabine chemotherapy. Currently, there are no clear guidelines for the management of bone marrow metastasis due to breast cancer, as most of the studies exclude these patients.

Response to therapy is also highly variable, as Sasada et al. reported; a patient with breast cancer with bone marrow metastasis later developed pancytopenia and disseminated intravascular coagulation [17]. This patient was managed with weekly paclitaxel therapy, granulocyte-colony stimulating factor injections, and blood transfusion, but therapy was ineffective, and the patient died due to gastrointestinal hemorrhage likely secondary to worsening pancytopenia. Our patient also had similar bone marrow metastasis with pancytopenia, but we were able to achieve disease control with capecitabine chemotherapy without any bleeding complication.

## Conclusions

Breast cancer commonly metastasizes to bone marrow, but it does not cause bone marrow failure. In this case, our patient’s initial presentation of breast cancer was pancytopenia which was diagnosed later due to bone marrow failure secondary to metastasis. This is an extremely rare clinical presentation. In addition, our patient showed a good clinical response to capecitabine chemotherapy, and her platelet count reached near baseline values. Therefore, this case report suggests physicians should consider that breast cancer is another differential for pancytopenia; if missed, pancytopenia can lead to devastating complications. Further research is needed regarding the proper management of breast cancer patients with bone marrow metastasis.
